# Structure and Validity of Questionnaire for Oral Frail Screening

**DOI:** 10.3390/healthcare9010045

**Published:** 2021-01-05

**Authors:** Yoshiaki Nomura, Yoshimasa Ishii, Yota Chiba, Shunsuke Suzuki, Akira Suzuki, Senichi Suzuki, Kenji Morita, Joji Tanabe, Koji Yamakawa, Yasuo Ishiwata, Meu Ishikawa, Kaoru Sogabe, Erika Kakuta, Ayako Okada, Ryoko Otsuka, Nobuhiro Hanada

**Affiliations:** 1 Department of Translational Research, Tsurumi University School of Dental Medicine, Yokohama 230-8501, Japan; ishikawa-me@tsurumi-u.ac.jp (M.I.); sogabe-k@tsurumi-u.ac.jp (K.S.); otsuka-ryoko@tsurumi-u.ac.jp (R.O.); hanada-n@tsurumi-u.ac.jp (N.H.); 2 Ebina Dental Association, Kanagawa 243-0421, Japan; ishiiryo141@gmail.com (Y.I.); yota@db3.so-net.ne.jp (Y.C.); shun-s@wg8.so-net.ne.jp (S.S.); suzuki@bell-dental.com (A.S.); lion@kd5.so-net.ne.jp (S.S.); morita-d-c-2@t06.itscom.net (K.M.); tanabedental5@me.com (J.T.); cherry@cherry-dental.com (K.Y.); yasuo-i@rb3.so-net.ne.jp (Y.I.); 3 Department of Oral Microbiology, Tsurumi University School of Dental Medicine, Yokohama 230-8501, Japan; kakuta-erika@tsurumi-u.ac.jp; 4 Department of Operative Dentistry, Tsurumi University School of Dental Medicine, Yokohama 230-8501, Japan; okada-a@tsurumi-u.ac.jp

**Keywords:** oral frailty, screening, validity, Item Response Theory

## Abstract

Oral frailty is defined as the mild decline in oral function and located at the early and reversible stage of frailty. Therefore, early detection and early treatment of oral frailty is very useful. Simple and easy questionnaires, such as an oral frailty checklist, have been widely used for the screening and enlightenment of oral frailty of the Japanese people. We evaluate the structure and validity of the oral frailty checklist. The questionnaire of oral frailty was distributed for the citizens more than 50 years old from December 2018 to January 2019. The structural validity of the questionnaire is analyzed by structural equation modeling (SEM). The characteristics of the items are analyzed by Item Response Theory (IRT). The data of 725 subjects (360 men, 359 women, 6 no answer, mean age 71.3 ± 9.05) are analyzed. The questionnaire consisted of three latent variables. Items of “Brushing teeth at least twice a day”, “Regular attendance of dental clinic”, and “Using denture”, had low discrimination ability. The questionnaire used in this study is a useful tool for the screening of oral frailty. However, its scoring system needs to be improved.

## 1. Introduction

The costs of medical healthcare cost and nursing care have remarkably increased in Japan because of the increase in older people requiring care [1,2,3]. The Japanese national insurance system covers the medical healthcare cost and nursing care cost of older people and a quarter of its expenses for the older people is borne by national assets. Therefore, increasing the medical and nursing cost is a critical national issue.

Frailty is assigned between health and disease or health and disability. Increasing attention has been paid to physical frailty as a risk of disability especially in older people. The negatively chained frailty cycle has been proposed [4]. Sarcopenia is located at the center of this cycle. Sarcopenia is defined as an age-related decline in lean body mass, muscle mass, and function [5,6]. Slightly progressed sarcopenia decreases metabolism and energy consumption. It leads to the decrease of appetite, then falls into malnutrition and weight loss, and promotes the progress of the next sarcopenia stage. Social issues such as single life, withdrawal, poverty, and psychological issues (cognitive impairment, depression, etc.) are also greatly involved in the progress of sarcopenia. The early breaking of this negative cycle is important. In this context, detecting the frailty of oral functions is reasonable. Oral health is related to appetite and malnutrition. It also related to social activity. Oral frailty is defined as the mild decline in oral function and located in an early and reversible stage of frailty [7]. Therefore, early detection and early treatment of oral frailty is very useful.

Prevention of oral frailty can reduce medical and nursing care costs. Early detection and early treatment of oral frailty are expected to be countermeasures for the medical and nursing care cost reduction. Therefore, the concept of oral frailty was widely introduced in Japan [8,9]. In addition, oral hypofunction is recognized as a disease. Its examinations, treatment, and maintenances have been introduced in the Japanese national insurance system [10]. Many Japanese dental clinicians have started to care about the oral frailty of older people. The Japan dental association and its branch associations have begun to propagate the concept.

On the basis of these concepts, a simple and easy questionnaire, such as the oral frail checklist, has been widely used for the screening and enlightenment of oral frailty for the Japanese people. However, as far as we are aware, there is no report that confirmed the validity of this oral frailty checklist. In this study, we check and evaluate the structure and validity of the oral frailty checklist to understand the usefulness of the oral frail checklist.

## 2. Materials and Methods

### 2.1. Setting

The questionnaire of oral frailty was distributed for the citizens of Ebina city, located near the capital of Tokyo. A booth was set up for the survey outside of city hall, a housing estate, and a sports center from December 2018 to January 2019. Before distribution, age was asked. The questionnaire was distributed to subjects more than 50 years old. In the booth, dental hygienists counted the number of remaining teeth under the supervision of dentists [11].

### 2.2. Questionnaire

The oral frailty checklist proposed by the Japan dental association was used [11]. The checklist consisted of 8 items: 1: Harder to eat hard food than half a year ago. (Difficult to eat hard food), 2: Sometimes, choked by tea or soup (Choking), 3: Do you use denture (Using denture), 4: Minding about oral dryness (Xerostomia), 5: Less frequent going out times than half a year ago (Less frequently going out), 6: Capable of chewing hard food like pickled radish or shredded and dried squid (Feasible to chew hard food), 7: Brushing teeth at least twice a day (Brushing teeth at least twice a day), 8: Attending dentist at least once a year (Regular attendance of dental clinic).

By the standard protocol, items 1, 2, and 3 are weighted as two points and others were one point. The screening criteria is defined by the sum of the scores: Low risk for 0–2 points, risk for 3 points, and high risk for more than 4 points.

### 2.3. Statistical Analysis

To investigate the latent variables and their correlations of the items in the oral frailty checklist, factor analysis and structural equation modeling was carried out [11,12,13,14,15,16]. The analysis was performed by SPSS ver 24.0 (IBM, Tokyo, Japan) and AMOS ver 24.0 (IBM, Tokyo, Japan). Under the Item Response Theory (IRT) approach, three parameter logistic models were applied. [12,13,14,15,17,18]. Item difficulty, item discrimination, item information were calculated. Item response curve and item information curves were graphically illustrated. Individual ability was calculated. Analysis of IRT were performed by R ver 3.50 with the ltm and irtoys packages. After checking the normality of distribution by the Kolmogorov-Simonov test, the Mann Whitney U test was applied for the number of remaining teeth against the item response. Kruskal Wallis tests were applied for the ability calculated by IRT analysis against the groups by number of remaining teeth. To visualize the response for each item and number of remaining teeth, correspondence analysis was carried out [15,17,19]. The analysis was performed by SPSS ver 24.0 (IBM, Tokyo, Japan).

## 3. Results

### 3.1. Structure of the Oral Frailty Screening Questionnaire

**Figure 1:**
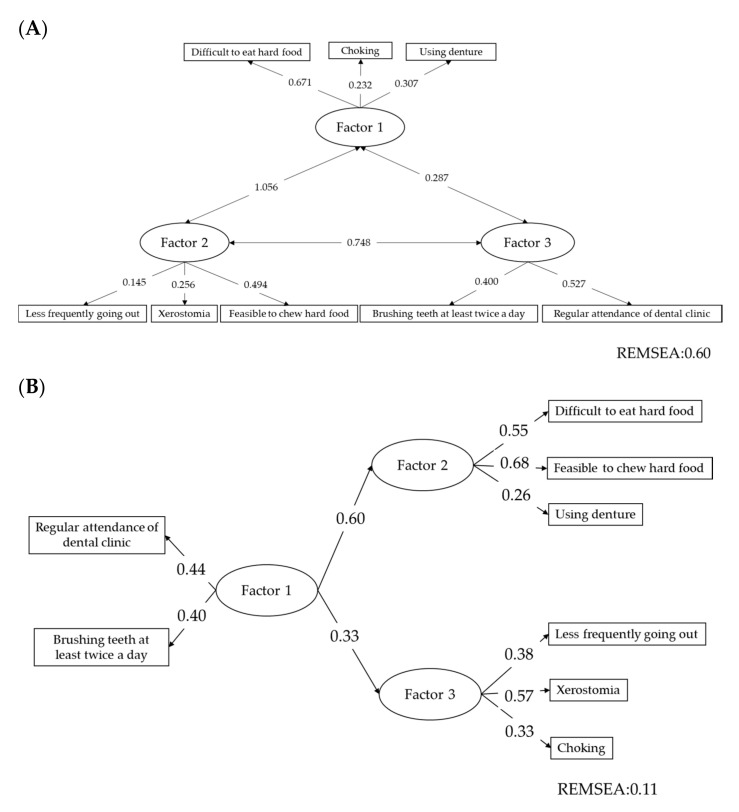
Path diagram of the items of the oral frailty screening questionnaire. (**A**) Default model. (**B**) Improved model. In the default model, the path between Factor 2 and 3 was not statistically significant. It was removed in the improved model. Fitness index REMSEA was improved when compared with the default model. All the paths were statistically significant. REMSEA: Root Mean Square Error of Approximation.

The distribution and collection of the questionnaire was conducted simultaneously. Therefore, all of the questionnaires were collected. The data of 725 subjects (360 men, 359 women, and 6 no answer, mean age 71.3 ± 9.05) were analyzed. The frequency of each item is shown in Table S1. The structural validity of the questionnaire was analyzed by factor analysis and structural equation modeling (SEM). The results of the factor analysis is shown in Table S2. By the varimax rotation, the questionnaire consisted of three latent variables. Using this classification, SEM was carried out. The default model is illustrated in Figure 1A. Then, the statistically non-significant paths between Factor 2 and 3 were removed. The improved model is illustrated in Figure 1B. The fitness index REMSEA was improved and all path were statistically significant. The path from Factor 1 to Factor 2 was 0.60 and from Factor 1 to Factor 3 was 0.33.

### 3.2. Item Response Theory Analysis of the Oral Frailty Screening Questionnaire

**Figure 2:**
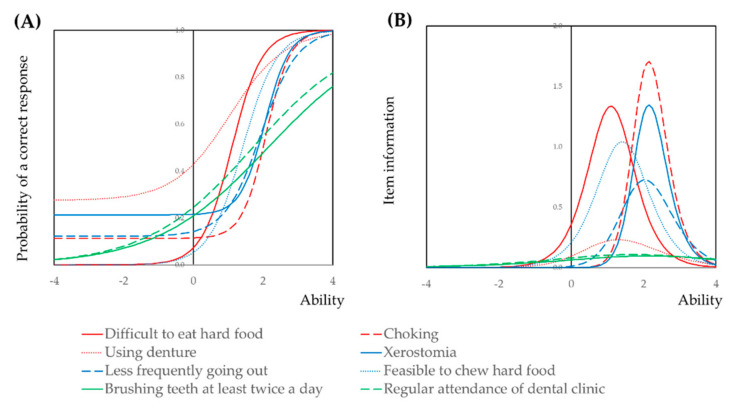
Item response curves and item information curves of items of the oral frailty screening questionnaire. Three parameter logistic models were applied separately by the latent variable shown in Table S2. The item response curve of the “Brushing teeth at least twice a day”, “Regular attendance of dental clinic”, and “Using denture,” are gentle. The item information of these items are low. The item response curves of “Difficult to eat hard food”, “Feasible to chew hard food”, are located in backward direction when compared to those of “Choking”, and “Xerostomia”.

The characteristics of each item of the questionnaire was analyzed by three parameter logistic model under the Item Response Theory approach (IRT). The model is shown in Table S3. The item response curves and item information curves are shown in Figure 2. The item response curve of the “Brushing teeth at least twice a day”, “Regular attendance of dental clinic”, and “Using denture,” are gentle. The item information of these items are low. The item response curves of “Difficult to eat hard food”, “Feasible to chew hard food”, are located in the backward direction when compared to those of “Choking”, and “Xerostomia”.

### 3.3. Effect of Number of Remaining Teeth on the Oral Frailty Screening Questionnaire

The correlation between number of reaming teeth and item response was analyzed. The ability calculated by IRT analysis and standardized scores of the questionnaire were not normally distributed by the Kolmogorov-Simonov tests. Data are expressed as the mean ± SD and median and 25th–75th percentile (Table 1). The differences of ability and scores against the categorized number of remaining teeth were statistically significant according to the Kruskal Wallis test. By the multiple comparison, the group of number of remaining teeth more than 25 had statistically significant differences in both scores and ability between all other groups. The group of number of remaining teeth 21−24 had statistically significant differences between all other groups. Other than that, there were no statistically significant differences both in ability and scores.

Summary statistics of number of remaining teeth against the eight items are shown in Table S4. Number of remaining teeth were significantly lower in subjects with “Feasible to eat hard food,” “Choking,” and “Using denture,” and lower in subjects with “Feasible to chew hard food,” and “Brushing teeth at least twice a day”.

**Figure 3:**
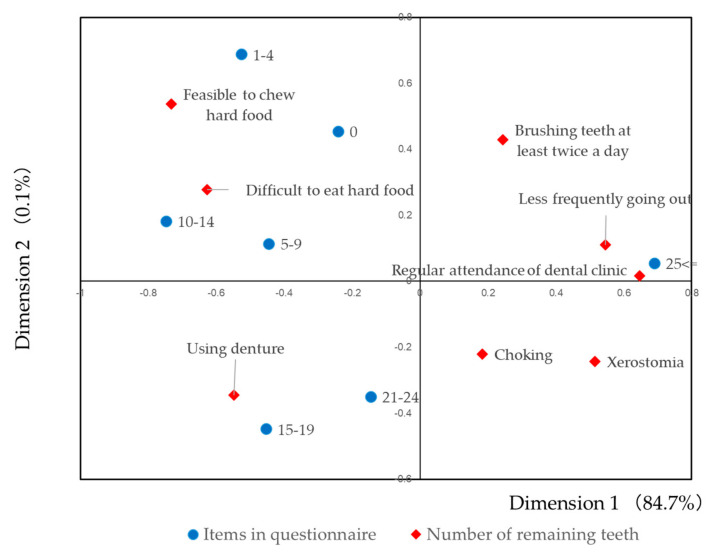
Correspondence analysis for the item response tendency. Subjects with high number of remaining teeth answered, “Regular attendance of dental clinic” and “Less frequently going out”. These behaviors may be the first stage of oral frailty. Subjects with small number of remaining teeth answered, “Feasible to chew hard food” and “difficult to eat hard food”. These mastication disabilities may be the progressed stage of oral frailty. Therefore, the levels of items were different by the number of remaining teeth.

To visualize the correlation between the response for each item and number of remaining teeth, correspondence analysis was carried out. The result is illustrated in Figure 3. The groups lower in number of remaining teeth were located around the items concerning chewing ability: “Feasible to eat hard food”, “Difficult to eat hard food”. Using denture was located near the groups of “15–19” and “21–24”. Items of “Choking,” and “Xerostomia” were separately located form other items.

## 4. Discussion

In this study, we analyzed the validity of the oral frailty checklist widely used in Japan [11]. The oral frailty checklist consisted of three latent variables. The three latent variables were very reasonable. “Attendance of dental clinic at least once a year” and “Brushing teeth at least twice a day” were designated as the background factors. These two items are oral health behaviors. Chewing ability and oral conditions for social relationship (going out, xerostomia and choking) were designated as the dependent variables. Among them, “Difficulty to eat hard food” and “Choking” had high discrimination ability by IRT analysis.

Several studies have tried to measure and evaluate the oral functions especially in older adults [20,21,22,23,24]. The oral function proposed by the Japanese national insurance system consisted of several dimensions [10]. The functions or symptoms such as tongue function, spilling foods, and slight choking were measured and evaluated in combination with questionnaires and specialized devices. Measuring oral function by using devices is time consuming and a burden on screening or on the use of public health facilities. The decrease in oral functions leads to sarcopenia, and this can be a risk of mortality of older subjects [25,26]. Simple questionnaires are more suitable for screening or for the use of public health facilities. Therefore, it is necessary to develop sophisticated questionnaires that represent the oral functions.

When analyzed eight items simultaneously by IRT, the items of “Regular attendance of dental clinic” and “Brushing teeth at least twice a day” had high discrimination ability. However, the item response curve of these two items descended with the increase of ability. Therefore, it is not reasonable to analyze all the items simultaneously. The items should be analyzed separately by their latent variables.

The cutoff point of the oral frailty screening questionnaire was set at weighted sum of the weighted score of the eight items. Two items “Difficult to eat hard food”, “Choking”, and “Using dentures” were weighted two and other items were one. More than four of the total score is considered to be a risk of oral frailty. Among the eight items “Difficult to eat hard food” and “Choking” had high discrimination ability by IRT. However, “Difficult to eat hard food “and “Using dentures” were derived from one latent variable, and “Choking” was derived from another latent variable. Oral function is multidimensional and oral frailty is also multidimensional [27]. A simple sum of the scores may not be appropriate for the screening of oral frailty. For the current questionnaire, the weight of the item can be improved by applying the item discriminations shown in Table S3. In addition, multidimensional evaluation based on the structure presented in Figure 1 is more suitable, including the recommendation for the prevention of oral frailty.

Characteristics of each item were visualized by correspondence analysis [15,17,19]. As shown in Figure 3, subjects with a high number of remaining teeth answered, “Regular attendance of dental clinic” and “Less frequently going out”. These behaviors may be the first stage of frailty, including oral frailty. Subjects with a small number of remaining teeth answered, “Feasible to chew hard food” and “Difficult to eat hard food”. These mastication disabilities may be the progressed stage of the oral frailty. Therefore, the levels of items were different by the number of remaining teeth. As shown in Table 1, dose response relationships were observed between the number of remaining teeth and total ability by IRT. This fact indicates that the questionnaire used in this study reflected the number of remaining teeth conditions. However, some of the items were not significantly correlated with the number of remaining teeth (Table S4). These factors effected oral frailty independently from the number of remaining teeth.

There are still several limitations of this study and the questionnaire. For the screening, sensitivity and specificity should be evaluated by using the gold standards. In this case, oral functions including tongue pressure and chewing efficiency should be evaluated simultaneously by using measuring instruments as an objective indicator. Halitosis in patients with remaining teeth should be measured for oral hygiene status. The cutoff point and weight of the items should be established by using ROC curves and statistical modeling. In addition, socioeconomic and education status were not evaluated. Even through there are life security systems for Japanese older adults such as pension or the national health insurance system, oral conditions may reflect the socioeconomic and education status during younger age. The evaluation of oral function is common to each country, however, the supply system of medical and dental service and the insurance system differ between countries. Therefore, the item of “Regular attendance of dental clinic” needs confirmation for its application in other countries. However, it is valuable information to confirm the structural validity of the currently and widely used questionnaires and characteristics of the items by a large number of people.

## 5. Conclusions

The questionnaire for the screening of oral frail is a validated, useful tool. However, the scoring system of the questionnaire should be established on the basis of IRT analysis.

## Figures and Tables

**Table 1:** Ability and scores by the oral frailty checklist of the groups by number of remaining teeth.

		Ability		Score	
Number of Remaining Teeth	*N*	Mean ± SD	Median (25th–75th)	Mean ± SD	Median (25th–75th)
0	34	0.84 ± 0.14	0.81 (0.22–1.34)	3.36 ± 0.37	3 (2–5)
1–4	19	0.97 ± 0.17	0.94 (0.22–1.48)	3.47 ± 0.34	3 (3–4)
5–9	37	0.72 ± 0.12	0.72 (−0.14–1.41)	3.05 ± 0.27	3 (2–5)
10–14	54	0.59 ± 0.09	0.63 (−0.14–1.15)	2.57 ± 0.18	2 (1–4)
15–19	62	0.43 ± 0.09	0.20 (−0.15–0.88)	2.36 ± 0.18	2 (1–3)
21–24	156	0.12 ± 0.05	−0.14 (−0.26–0.54)	1.75 ± 0.11	1 (1–2)
25≤	48	−0.13 ± 0.03	−0.15 (−0.51–0.01)	1.32 ± 0.06	1 (0–2)

The ability and scores were not normally distributed by Kolmogorov-Simonov tests. Data are expressed as the mean ± SD and median and 25th−75th percentile. The differences of ability and scores of the groups were statistically significant according to the Kruskal Wallis test. By the multiple comparison, the group of number of remaining teeth more than 25 had statistically significant differences in both scores and ability between all other groups. The 21–24 group had statistically significant differences between all other groups. Other than that, there were no statistically significant differences both in ability and scores.

## Data Availability

Data is available from the corresponding author by reasonable request.

## Supplementary Materials

The following are available online at https://www.mdpi.com/2227-9032/9/1/45/s1. Table S1: Descriptive statistics of the data analyzed in this study, Table S2: Results of factor analysis of the oral frailty checklist, Table S3: Three parameter logistic model under Item Response Theory approach for the oral frailty checklist, Table S4: Number of remaining teeth against the response for the items in oral frailty checklist.
