# Supplementary material for: Structure and Validity of Questionnaire for Oral Frail Screening
Source: Healthcare (Basel). 2021 Jan 5;9(1):45. doi: 10.3390/healthcare9010045 (PMC7824860; doi:10.3390/healthcare9010045)
Supplement: Supplementary file 1 [file healthcare-09-00045-s001.pdf]

healthcare MDPI

Supplemental materials

Table 1. Descriptive statistics of the data analyzed in this study.

		Sex				Age		
		Men	Women	Total	P-value*	Mean	Median (25th -75th)	P-value**
Difficult to eat hard food	No	285	296	585	0.201	70.9 +/- 9.0	71 (64 - 78)	0.045
	Yes	75	61	138		73.0 +/- 9.3	73 (68 - 78)	
Choking	No	300	311	616	0.209	71.0 +/- 9.1	71 (64 - 78)	0.040
	Yes	58	46	105		73.3 +/- 8.8	74 (67 - 78)	
Using denture	No	175	203	382	0.035	70.0 +/- 9.1	70 (63 - 77)	<0.001
	Yes	181	153	336		72.9 +/- 8.8	74 (68 - 79)	
Xerostomia	No	279	262	546	0.123	71.1 +/- 8.9	71 (64 - 78)	0.265
	Yes	79	97	177		72.0 +/- 9.6	73 (66 - 78)	
Less frequently going out	No	288	294	586	0.565	70.3 +/- 8.7	70 (64 - 77)	<0.001
	Yes	70	64	136		75.6 +/- 9.5	76 (70 - 82)	
Feasible to chew hard food	Yes	310	306	621	0.879	70.8 +/- 9.1	71 (64 - 78)	<0.001
	No	50	51	102		74.8 +/- 8.3	76 (69 - 81)	
Brushing teeth at least twice a day	Yes	251	304	560	<0.001	71.1 +/- 8.9	71 (65 - 78)	0.119
	No	108	54	163		72.2 +/- 9.5	73 (66 - 79)	
Regular attendance of dental clinic	No	252	275	531	0.037	71.5 +/- 8.6	72 (66 - 78)	0.419
	Yes	106	81	189		70.7 +/- 10.1	70 (63 - 78)	

*: P-values were calculated by chi-square tests. **: Age was not normally distributed by Kolmogorov Simonov tests (P<0.001), P-values were calculated by Mann-Whitney's U test.

Table 2. Results of factor analysis of the oral frailty check list.

	Factors		
	1	2	3
Difficult to eat hard food	0.54	0.19	0.17
Choking	0.51	0.12	0.27
Using denture	0.43	0.01	-0.07
Xerostomia	-0.09	0.74	0.01
Less frequently going out	0.10	0.34	0.11
Feasible to chew hard food	0.14	0.28	-0.03
Brushing teeth at least twice a day	-0.07	0.09	0.76
Regular attendance of dental clinic	0.14	<0.01	0.27
Total	0.80	0.79	0.78
% of variance	9.97	9.87	9.69
Cumulative %	9.97	19.84	29.52

Factor analysis was carried out by most likelihood method with varimax rotation.

Table 3. Three parameter logistic model under item response theory approach for the oral frailty check list.

Factor	Item	Discrimination	Difficulty	Guessing
1	Difficult to eat hard food	2.31	1.10	<0.01
	Choking	2.91	2.08	0.11
	Using denture	1.25	1.04	0.27
2	Xerostomia	2.85	2.05	0.21
	Less frequently going out	1.92	1.94	0.12
	Feasible to chew hard food	2.04	1.41	<0.01
3	Brushing teeth at least twice a day	0.62	2.14	<0.01
	Regular attendance of dental clinic	0.66	1.71	<0.01

Table 4. Number of remaining teeth against the response for the items in oral frailty check list.

			**Number of remaining teeth**		
**Item**	**Score**	n	Mean +/- SD	Median(25th-75th)	P-value
Difficult to eat hard food	0	585	22.45+/-0.3	25(21-28)	<0.001
	2	138	15.07+/-0.79	16(7-23)	
Choking	0	616	21.36+/-0.32	25(18-27)	0.023
	2	105	19.17+/-0.89	22(13-26)	
Using denture	0	382	25.28+/-0.25	27(24-28)	<0.001
	2	336	16.29+/-0.47	18(10-24)	
Xerostomia	0	546	21.29+/-0.34	25(18-27)	0.236
	1	177	20.41+/-0.66	24(16-27)	
Less frequently going out	0	586	21.39+/-0.32	24(18-27)	0.351
	1	138	19.74+/-0.83	24(14-27)	
Feasible to chew hard food	1	621	13.95+/-0.92	14(6-22)	<0.001
	0	102	22.25+/-0.3	25(20-27)	
Brushing teeth at least twice a day	1	560	18.44+/-0.78	23(11-27)	0.001
	0	163	21.81+/-0.32	25(19-27)	
Regular attendance of dental clinic	1	531	20.23+/-0.66	25(17-27)	0.270
	0	189	21.39+/-0.34	24(18-27)	

P-values were calculated by Mann Whitney's U tests. There existed several missing values for the item response.
